# Draft Genome Sequence of Root-Associated Sugarcane Growth-Promoting *Microbispora* sp. Strain GKU 823

**DOI:** 10.1128/genomeA.00647-17

**Published:** 2017-07-20

**Authors:** Worarat Kruasuwan, Paul A. Hoskisson, Arinthip Thamchaipenet

**Affiliations:** a Department of Genetics, Faculty of Science, Kasetsart University, Chatuchak, Bangkok, Thailand; b Strathclyde Institute of Pharmacy and Biomedical Sciences, University of Strathclyde, Glasgow, United Kingdom; c Center for Advanced Studies in Tropical Natural Resources, NRU–KU, Kasetsart University, Chatuchak, Bangkok, Thailand

## Abstract

The endophytic plant growth-promoting *Microbispora* sp. strain GKU 823 was isolated from the roots of sugarcane cultivated in Thailand. It has an estimated 9.4-Mbp genome and a G+C content of 71.3%. The genome sequence reveals several genes associated with plant growth-promoting traits and extensive specialized metabolite biosynthesis.

## GENOME ANNOUNCEMENT

Endophytic actinomycetes are free-living filamentous bacteria that mutually colonize inside plants and facilitate plant growth through direct and indirect mechanisms employing plant growth-promoting (PGP) traits (1). The endophytic *Microbispora* sp. strain GKU 823 was isolated from roots of sugarcane cultivated in Thailand and has been shown to enhance sugarcane growth (2). This strain exhibits PGP traits, including the production of indole-3-acetic acid (IAA) and siderophores, phosphate solubilization, and suppression of fungal growth (2). Based on 16S rRNA gene sequence analysis, *Microbispora* sp. GKU 823 is closely related to *Microbispora hainanensis* 211020^T^ (99.09% similarity, GenBank accession no. KR560040). The whole-genome sequence of *Microbispora* sp. GKU 823 was obtained, and genes associated with plant growth promotion and specialized metabolite biosynthesis were identified.

Total genomic DNA of *Microbispora* sp. GKU 823 was extracted using the Isolate II genomic DNA extraction kit (Bioline, UK), according to the manufacturer’s instruction. The genome was sequenced using the Ion PGM system, generating 1,230,781 reads (with approximately 30× coverage) with an average read length of 225 bp. The genome was assembled using SPAdes version 3.9 (3) and evaluated by QUAST version 3.2 (4). Reads were assembled into 262 contigs (coverage, ≥10×; length, ≥1,000 bp), with an *N*_50_ of 69,483 bp. The largest contig obtained was 321,219 bp in length. The draft genome is estimated to be 9,430,099 bp, with a G+C content of 71.3%.

Functional gene annotation of the assembled genome was carried out by the Rapid Annotations using Subsystems Technology (RAST) server (5). rRNA and tRNA genes were determined by RNAmmer (6) and tRNAscan-SE (7), respectively. The annotation predicted a total 9,248 coding sequences, 58 tRNA genes, and 3 rRNA genes. The average nucleotide identity values of the genome by BlastN (ANIb) were calculated in JSpeciesWS (8). The genome comparison revealed that *Microbispora* sp. GKU 823 had an ANIb value of 92.47% similar to *Microbispora rosea* NRRLB-2630, 90.70% to *M. rosea* NRRL B-2631, and 81.20% to *Microbispora* sp. strain ATCC PTA-5024.

Genes related to PGP traits, including phosphate solubilization (alkaline phosphatase and isocitrate dehydrogenase [9, 10]), indole-3-acetic acid (IAA) production (tryptophan 2-monooxygenase [11]), and genes involved in fungal cell wall degradation (family 18 chitinase [12]) were detected in the genome of *Microbispora* sp. GKU 823. Moreover, genes involved in stress tolerance (betaine aldehyde dehydrogenase, proline dehydrogenase, superoxide dismutase, and trehalose synthase [13]) were also present. These genes may provide *Microbispora* sp. GKU 823 with the capability to promote the growth of sugarcane (2). antiSMASH version 3.0 (14) predicted 23 secondary metabolite gene clusters in the genome of *Microbispora* sp. GKU 823, including seven gene clusters of nonribosomal peptide synthetase, four gene clusters of type I polyketide synthase and terpene, three gene clusters of bacteriocin, two gene clusters encoding siderophores (including desferrioxamine E), and a single gene cluster encoding a lanthipeptide. These secondary metabolite gene clusters indicate that endophytic *Microbispora* species are potential sources of novel specialized metabolites.

### Accession number(s).

The draft genome sequence of *Microbispora* sp. GKU 823 has been deposited in the DDBJ/ENA/GenBank databases under the accession number MWJN00000000.
